# Pediatric patient fixation for air aeromedical transport in rotary-wing aircraft: a professional experience report

**DOI:** 10.1590/1980-220X-REEUSP-2026-0017en

**Published:** 2026-07-24

**Authors:** Euseli de Assis Batista, José Colleti

**Affiliations:** 1 Hospital lsraelita Albert Einstein, São Paulo, SP, Brazil.

**Keywords:** Aircraft, Air Ambulances, Emergencies, Pediatrics, Guidelines as Topic.

## Abstract

**Objective:**

To report on the experience of developing a care protocol for pediatric patients fixation for the transflight in rotary-wing aircrafts.

**Method:**

Professional experience report. The protocol was developed within the aeromedical urgency and emergency service of Santa Catarina, guided by the Guide for the Construction of Care Protocols of Coren/São Paulo. The theoretical framework was based on an integrative review and consultation of documents from national and international official agencies, as well as the Brazilian Federal Nursing Council. Internal validation was carried out with eight flight nurses from the service.

**Results:**

The protocol structured the care for securing the pediatric patient into three care phases: preflight, transflight, and postflight, supporting clinical decision-making and safety during transport.

**Conclusion:**

The developed protocol standardized the practices for pediatric patients fixation in aeromedical services, promoting safety and supporting clinical decision-making during transflight, minimizing the risk of complications during transport, and strengthening professional practice in the field of aeromedical services.

## INTRODUCTION

Aeromedical transport consists of the rescue or removal of individuals affected by serious health conditions, carried out using fixed-wing or rotary-wing aircrafts, integrated into the pre-hospital care system and the medical emergency regulation center^(1)^.

Aeromedical emergency services using rotary-wing aircrafts are guided by international recommendations from the Air Medical Physicians Association, the National Association of EMS Physicians, and the American College of Surgeons Committee on Trauma^(2)^. In Brazil, the aeromedical service is regulated by Ordinance No. 2048/2002 of the Ministry of Health for the exercise of the professional activity in type E ambulances, comprising fixed-wing and rotary-wing aircrafts regulated according to the standards of the National Civil Aviation Agency^(3)^. Regarding professionals working in aeromedical services, the nurse is responsible for the assistance and management of mobile pre-hospital and inter-hospital care in an aircraft, as regulated by Resolution No. 660/2021 of the Federal Nursing Council^(4)^.

Aeromedical transport by rotary-wing aircraft covers all age groups, providing rapid care to patients with potentially life- threatening acute conditions, cardiac, neurological or vascular emergencies, severe trauma, and accidental injuries^(5,6,7)^.

This type of transport, used for pediatric patients, is indicated for those who are seriously ill or injured, generally identified by the air ambulance team in the pre-hospital period, resulting in triage for tertiary emergency services^(8,9)^. In the pediatric context, the anatomical and physiological particularities of the pediatric patient increase vulnerability to trauma, such as the disproportion between head and body in early childhood, which raises the frequency of falls and the risk of injuries, including head trauma^(10)^. Furthermore, the smaller body mass and the proximity between organs favor greater energy dissipation during impacts due to skeletal flexibility, increasing the likelihood of systemic injuries and favoring the occurrence of visceral injuries without evident external signs^(10)^.

Moreover, pediatric patients are more vulnerable to hypothermia when they suffer trauma^(10)^. Regarding the airways, they have a relatively larger tongue and a smaller oral cavity, increasing the risk of airway obstruction by foreign bodies (choking) due to exploratory behavior up to three years of age, or even obstruction due to tongue relaxation in cases of unconsciousness^(10)^.

Therefore, the clinical assessment of pediatric patients represents a significant challenge for the air ambulance team. A Danish cohort study reported that 6.4% of pediatric emergencies attended by the service, initially assessed as “non-critical,” require https://www-sciencedirect-com.ez46.periodicos.capes.gov.br/science/article/pii/S1067991X25001579?via%3Dihub#bib0007intensive care within the following 24 hours^(5)^. In light of the above, it is important to highlight that aeromedical transport is fundamental in managing urgent and emergency situations, especially in conditions with geographical barriers or long distances from the incident^(11)^.

However, this type of transport presents operational and care challenges due to the environmental conditions of the flight, aircraft space, vibrations, noise, and adaptation for performing clinical procedures in an air environment^(12)^. Therefore, teams caring for/transporting children must be prepared with the full spectrum of equipment sizes necessary for securing pediatric patients, who range from premature infants (< 500 grams) up to a child weighing 100 kg or more^(13)^.

In this framework, based on professional practice as a flight nurse in aeromedical transport, a gap related to the lack of standardization for the safe securement of pediatric patients in rotary-wing aircraft was identified. The literature review confirmed the scarcity of specific protocols aimed at this purpose^(14)^. This is corroborated by the absence of international guidelines regulating the standard of care during aeromedical transport^(15)^ and by the variability in pediatric restraint practices identified in the literature^(16)^. This study aimed to report on the experience of developing a care protocol for pediatric patients securement for transflight in rotary-wing aircrafts.

## METHOD

### Design of Study

This is a professional experience report study, which describes the development of a nursing care protocol aimed at securing pediatric patients during aeromedical transport in rotary-wing aircrafts.

### Local

The protocol was developed and implemented in the aeromedical urgency and emergency service, within the context of the Brazilian Mobile Emergency Service (*SAMU*) of Santa Catarina, where it is known as *Arcanjos*. The *Arcanjo* service operates with two helicopters - the *Arcanjo* 01 rotary-wing Squirrel helicopter, based in Florianópolis, and the other, Arcanjo 03, based at Blumenau airport. Rotary-wing *Arcanjos* come in different configurations, regarding crew and type of operation, and can be classified as high-risk or high-complexity. Based on the incident, the aircraft crew may consist of two pilots (dual command), an operational crew member, a doctor, and a flight nurse. In high-risk or highly complex operations, a sixth operational rescue crew member may be added.

### Population

The protocol developed covers pediatric patients attended by the aeromedical urgency and emergency service.

### Data Collection

The protocol development was based on the Guide for the Construction of Care Protocols from the Regional Nursing Council of São Paulo^(17)^. This guide provides sequential steps for building protocols, including: defining the protocol theme and scope, conducting a literature review and reviewing scientific evidence, developing content and flowcharts, internally validating with the professionals involved, and implementing the service.

For the theoretical foundation, an integrative literature review was conducted in the following databases. Medical Literature Analysis and Retrieval System Online, Cumulative Index to Nursing and Allied, Scopus, Latin American and Caribbean Literature in Health Sciences, Embase; Nursing Database and Scientific Electronic Library Online, where the Health Sciences Descriptors and Medical Subject Headings were used: *“Ambulância aérea”, “Serviço aeromédico”, “Paciente pediátrico”, “Helitransportados”, “Helicóptero de emergência”, “Ambulância aérea”, “Resgate aéreo”, “Atendimento de emergência pré-hospitalar”.* (“Air ambulance”, “Aeromedical service”, “Pediatric patient”, “Helicopter transported”, “Emergency helicopter”, “Air ambulance”, “Air rescue”, “Pre-hospital emergency care”).The search covered the period from 2010 to 2022, initially resulting in 412 articles. After applying the inclusion and exclusion criteria, reading titles and abstracts, and the full text, the sample consisted of four studies addressing aeromedical transport of pediatric patients and aspects related to safety.

Additionally, regulatory documents from the Ministry of Health and national and international official agencies were consulted, as well as Resolutions from the Federal Nursing Council, with the aim of incorporating legal and technical- assistance recommendations that guide professional practice in aeromedical services. Based on the identified scientific and normative evidence, the protocol was structured to include pediatric patient restraint care in three phases: preflight care, transflight care related to pediatric patient restraint, and postflight care.

The internal validation phase of the protocol was carried out with eight flight nurses working in the aeromedical urgency and emergency service in Santa Catarina. Following internal validation, the protocol was implemented in the aeromedical service as a guiding tool for clinical practice, being used in the aeromedical transport of pediatric patients, aiming at standardizing securement procedures and supporting clinical decision-making during care. This experience report details the construction of the protocol specifically, but does not detail the steps of internal validation and implementation in the aeromedical urgency and emergency service.

### Ethical Aspects

Since this is a report of professional experience, there was no need to submit it to the Research Ethics Committee (CEP), in accordance with CNS Resolution No. 510/2016.

## RESULTS

The experience resulted in the development a care protocol for pediatric patients securement for the transflight in a rotary- wing aircraft. This was structured based on scientific evidence, national and international regulations, and the clinical practice of the aeromedical service, and was validated internally by flight nurses.

The protocol structured the care for pediatric patient fixation into three care phases: preflight, transflight, and postflight. This organization allows systematizing procedures, reducing variability in clinical practice, and supporting the team’s decision- making in highly complex settings.

### Preflight Care

Preflight care includes explaining the responsibilities of nursing and medical professionals, as well as aircraft models and crew composition.

It is the nurse’s responsibility to check the equipment operation, materials availability and expiration dates, as well as the replenishment of supplies used during care, ensuring readiness for pediatric aeromedical transport. It is also their responsibility to participate, along with the physician, in decision-making regarding transportation, to complete and document the progress of patients treated, and, at the end of care, to clean the equipment and send the materials for disinfection^(3,4)^.

The medical team is responsible for checking the equipment operation, the availability of materials in the backpack and the replenishment of supplies used; completing patient progress notes after each incident; and prescribing controlled medications administered during care^(4)^.

Regarding aircraft models, these include the Squirrel model (HB – 350 B1), registration prefix PT, named “Helicopter Arcanjo 01” with capacity to transport six people, including two pilots, one operational crew member, one doctor, one nurse and one patient, not equipped with an aeromedical kit. The Squirrel model (AS-350 B2), registration prefix PR-BNU, named “Helicopter Arcanjo 03”, has similar capabilities, but with an aeromedical kit available for medical services.

### Care During Transflight Related to Pediatric Patient Fixation

During the call for care, information is obtained regarding the child’s medical history, age, and possible pathologies. The team checks the availability of equipment for performing victim extraction maneuvers, tests the operation of each device, and prepares the aircraft with materials and equipment, organizing them in a way that does not compromise assistance during the transflight. Together with the doctor, the equipment, materials, and medications are organized in a way that facilitates quick and safe access, ensuring the safe removal of the child^(3)^.

Regarding the equipment available, the service includes a rigid transport stretcher and adult and pediatric Kendrick Extrication Device (KED). The standardized weight configuration and suggested usage are designed to ensure the comfort of the child being transported. It should be noted that there are children whose heights do not correspond to their chronological age, and the suitability of the size of the equipment used is assessed at the time of service^(4)^. The rigid stretcher used as a transport support device has a weight capacity of up to 180 kg and allows for the secure fixation of the pediatric patient, and for examinations and procedures to be performed without the need for immediate removal of the device. It has black, red, green, and yellow harnesses^(4)^.

**Figure 1:**
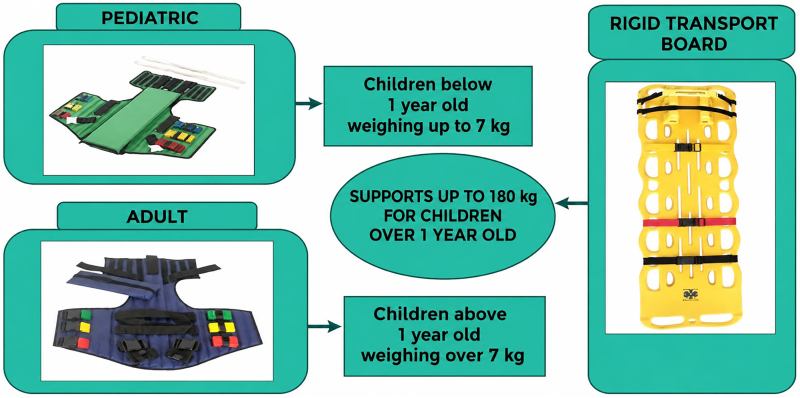
Equipment for transportation. Florianópolis, SC, Brazil, 2025.

The adult or pediatric KED consists of a dorsal immobilization vest associated with a rigid board, and is indicated for trauma victims who are seated, representing an option for transporting children in pre-hospital care. The rigid immobilization board is designed for transporting people weighing up to 180 kg. Both devices have a radiolucent structure, nylon straps with quick-release buckles, hand grips, a chin and forehead protection strap, pads, and quick-release straps in standard colors (yellow, green, red), allowing their use during examinations when necessary^(4)^ (Figure 1).

**Figure 2:**
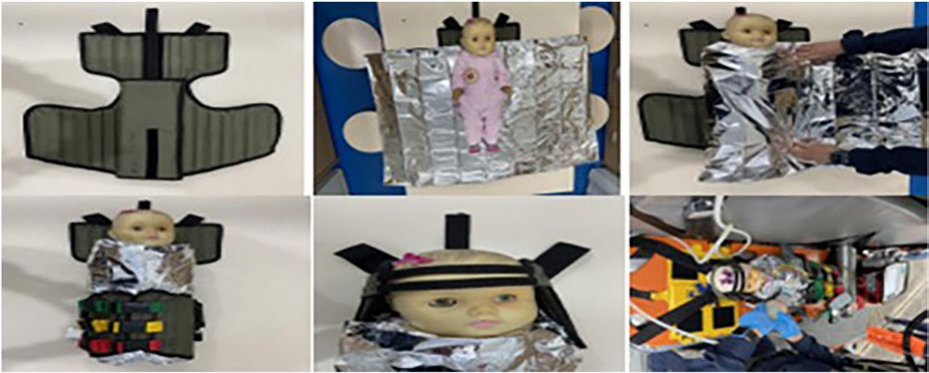
Fixation of the pediatric patient with Kendrick Extrication Device. Florianópolis, SC, Brazil, 2025.

When immobilizing a child for transport in an appropriately sized KED, the use of a thermal blanket is considered essential for controlling body temperature. The securement sequence initially prioritizes the head, followed by the torso and limbs. In the anatomical position, the empty spaces are filled with cushions, or by elevating the child’s torso when indicated. It is important to note that the securement must be carried out in a way that ensures stability and safety, without compromising respiratory movements (Figure 2).

Support for transport using a rigid stretcher completes the safety sequence during transflight, taking into account the injuries and/or clinical condition presented, allowing for adequate assistance during transport. The equipment features spider-straps, contributing to greater stability for the child during flight.

Children should not be carried on an adult’s lap during transflight due to the risk of falls or injuries, especially during turbulence or aircraft maneuvers. Securing the child in the aircraft seat allows for better clinical observation by the team, including assessment of skin tone, occurrence of seizures, vomiting, or behavioral changes, enabling chest compressions to be performed until an emergency landing is necessary.

The decision to discontinue immobilization, when necessary for patient safety, must be properly documented, with a detailed description of the reason. It is important to emphasize that immobilization should not impede ventilation, airway opening, or the performance of resuscitation maneuvers. In specific situations, it may be more appropriate to transport the child in their own car seat, when this proves to be safer. Children who react strongly to restraint attempts may be at greater risk of worsening spinal injuries; in these cases, alternative strategies are considered, such as distraction or guidance to remain still without rigid restraint^(3,10)^.

Prior to transflight, patient care priorities must be assessed and systematized, including reviewing all necessary equipment, ensuring secure fixation, and clearly and objectively documenting any incidents that occur during the preflight period^(3,4)^. Given the inherent risks of transflight, precautions related to securing the pediatric patient are essential to prevent the clinical condition from worsening. Securing the child to the aircraft seat promotes patient safety and allows the team to identify clinical changes early, such as seizures, vomiting, or changes in skin color. The combination of the KED straps, the rigid stretcher’s spider straps, and the aircraft’s harnesses provides greater stability, minimizing abrupt movements during transflight and contributing to the pediatric patient’s physical and mental well-being^(4)^.

During transflight, the doctor and nurse kneel on the aircraft floor due to the limited physical space and mobility constraints. Therefore, advanced life support procedures, such as orotracheal intubation, chest compressions, cardiac defibrillation, and chest drainage, are planned prior to boarding.

The protocol establishes that the transflight of a pediatric patient without mechanical ventilation should be carried out through the left door of the aircraft, entering in the cephalic position until complete caudal positioning. The choice of the left door in this situation is justified by the aircraft’s internal configuration, which favors positioning the patient’s head towards the healthcare team, allowing better access to the airways and monitoring during the flight. After boarding, the harnesses are fastened and the rigid board is anchored, along with the necessary equipment, according to the child’s age and weight. In children weighing approximately 7 kg and under 1 year of age, the protocol recommends the use of a pediatric KED. For children older than one year and weighing more than 7 kg, the protocol indicates the use of the adult device, along with a rigid board, for transfer to the aircraft^(4)^.

For pediatric patients on mechanical ventilation, the protocol dictates that entry in the transflight should occur through the aircraft right door, from the caudal position into full cephalic entry. This procedure is justified by the need to position the patient with their head close to the ventilation equipment, facilitating access to and control of the devices during the flight. It is important to note that the choice of access door may require adaptations depending on the landing site and terrain conditions, and it is necessary to assess, in each situation, whether the patient should be brought into the aircraft with their head or feet facing the entrance. After getting settled in, the safety harnesses are fastened and the rigid board is anchored to the crew seat, while the monitoring equipment is installed. In these cases, the use of an adult or pediatric KED, combined with a rigid backboard, promotes safety during transport and provides support for the accommodation of ventilation and monitoring equipment, enhancing the patient’s stability during the flight^(4)^.

### Postflight Care

At postflight, both the doctor and the nurse record and relay all information and incidents that occurred before, during, and after the child’s arrival at the treatment destination. The supplies and equipment used during the transfer of the pediatric patient are replenished, and the interior of the aircraft and the securing devices used in the transflight are cleaned and disinfected, ensuring the service is ready for future operations^(3,4)^.

**Figure 3:**
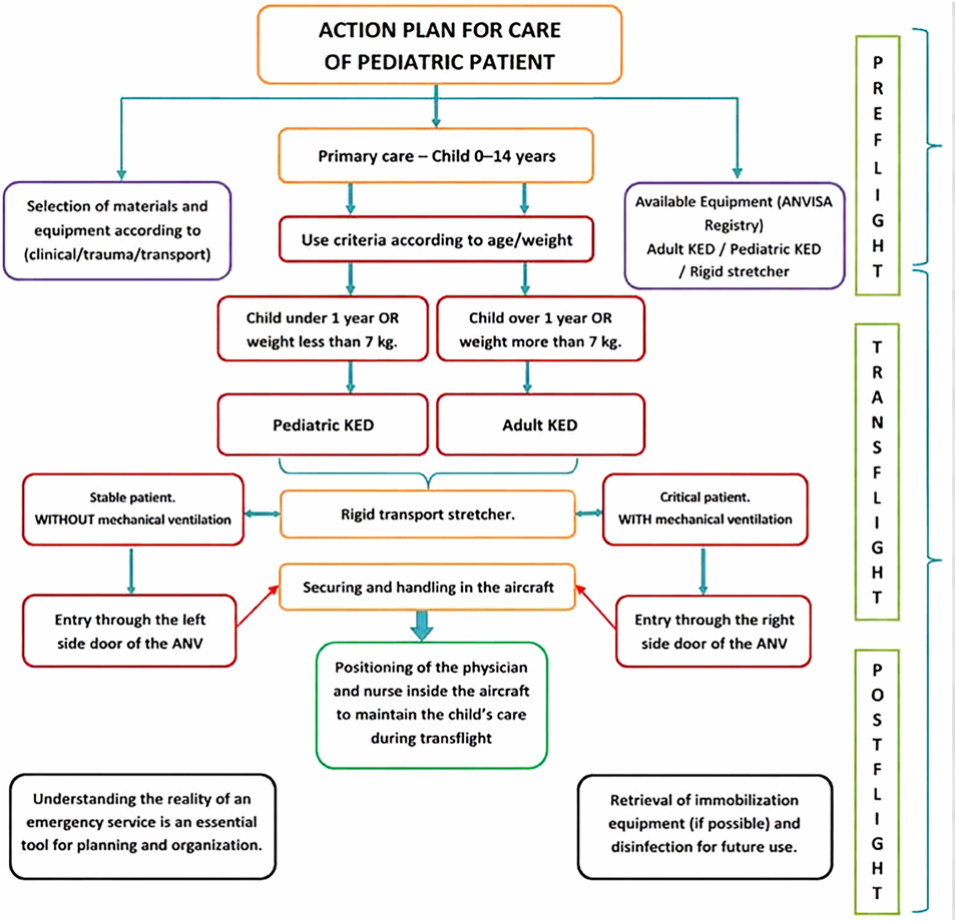
Flowchart of call for the care of a pediatric patient. Florianópolis, SC, Brazil, 2025.

The protocol also includes a care flowchart for pediatric patients, describing the steps corresponding to each phase of care during aeromedical transport (Figure 3).

## DISCUSSION

Aeromedical transport of pediatric patients in a rotary-wing aircraft is a highly complex healthcare practice, in which patient safety depends directly on the adequacy of the restraint devices and the organization of care provided during all stages of the flight. International studies show that variability in immobilization and securement practices during air transport can increase the risk of adverse events, especially in pediatric patients with unstable clinical conditions or on mechanical ventilation^(16,18)^.

Against this background, the reported experience demonstrates that the development of a specific care protocol for the fixation of pediatric patients contributed to the standardization of procedures and the reduction of variability in clinical practice within the aeromedical service. The organization of care occurred in three phases: preflight, transflight, and postflight, which favored advance planning, risk mitigation, and continuity of care, aspects considered essential in scenarios characterized by physical restraints, vibration, noise, and limited access to the patient during the flight^(2,7)^.

In the preflight phase, defining criteria for checking equipment and selecting securement devices, based on the child’s age, weight, height, and clinical condition, proved fundamental for the safety of the transport. Literature suggests that inadequate restraint devices for pediatric patients are associated with greater instability during transport and difficulty in performing emergency interventions^(19)^. Thus, systematizing this step contributes to the anticipation of healthcare needs and to more assertive clinical decision-making.

During transflight, the standardization of the application of fixation devices, prioritizing stability, airway maintenance, and the possibility of continuous monitoring, is supported by international recommendations for pediatric aeromedical transport. The anatomical and physiological specificities of this population, with a larger head size, skeletal flexibility, and greater vulnerability to hypothermia, require immobilization strategies that ensure safety without compromising ventilation or airway access^(10)^.

The use of KED in conjunction with a rigid board, as described in the protocol, proved to be consistent with these recommendations, providing adequate stability and support for necessary interventions during the flight. Another relevant aspect concerns the possibility of using alternative restraint devices, such as a car seat, in specific situations. Evidence suggests that, when properly secured, these devices can represent a safe alternative in aeromedical transport, especially for children who exhibit strong reactions to rigid immobility^(16)^.

In the postflight phase, standardizing the recording of care information and reorganizing the service’s operations contributes to continuity of care and patient safety, as well as to promoting traceability of the actions taken. The literature highlights that systematized records are essential for communication between teams and for evaluating the quality of care in emergency services^(3)^.

Also noteworthy is the role of the flight nurse as a central professional in organizing care and managing resources during aeromedical transport. The nurse’s role in checking equipment, applying securement devices, and making shared decisions with the medical team is in line with the regulations of the Federal Nursing Council, which recognizes their responsibility in both care and management in pre-hospital and inter-hospital air ambulance services^(4)^. Studies underscore the importance of preparing healthcare professionals, as performing life-saving procedures on children without the proper tools or training represents a significant challenge for the team^(20,21)^.

In light of the above, the protocol contributes to the standardization of pediatric patient securement during flight, strengthening professional autonomy and supporting clinical decision-making in highly complex settings where time and space impose significant limitations on care. One limitation of the study is the scarcity of scientific publications specifically focused on nursing practice in securing pediatric patients during aeromedical transport.

As a contribution to the healthcare field, the developed protocol describes the main securement procedures during the transflight of pediatric patients, gathering essential information for patient safety and showing potential applicability in other aeromedical contexts.

## CONCLUSION

Aeromedical transport of pediatric patients in rotary-wing aircraft exposes patients to inherent risks that can significantly interfere with their health status, requiring systematized preventive and therapeutic measures to minimize complications during transport. The reported experience demonstrates that developing a care protocol focused on pediatric patients securement contributes to improving the quality of care by systematizing procedures and reducing variability in care within a highly complex context. The developed protocol standardized the practices for pediatric patients securement in aeromedical service *Arcanjo*, promoting standardization, safety and support to clinical decision-making during transflight, minimizing the risk of secondary complications during transport, and strengthening professional practice in the field of aeromedical services.

## Data Availability

As recommended by Open Science, the link to the repository where the research data was deposited is: https://repositorio.ufsc.br/xmlui/bitstream/handle/123456789/247267/PGCF0174-D.pdf?sequence=1.

## References

[1] Sociedade Brasileira de Medicina Aeroespacial (2022). Transporte aeromédico [Internet].

[2] Lyng JW, Braithwaite S, Abraham H, Brent CM, Meurer DA, Torres A (2021). Appropriate air medical services utilization and recommendations for integration of air medical services resources into the EMS system of care: a joint position statement and resource document of NAEMSP, ACEP, and AMPA. Prehosp Emerg Care.

[3] Brasil. Ministério da Saúde. Portaria Nº 2048, de 5 de novembro de 2002 (2002). Regulamento técnico dos sistemas estaduais de urgência e emergência [Internet].

[4] Brasil. Conselho Federal de Enfermagem. Resolução Cofen nº 660/2021 que normatiza a atuação do enfermeiro na assistência direta e no gerenciamento do Atendimento Pré-hospitalar Móvel e Inter-hospitalar em veículo aéreo (2021). Diário Oficial da União [Internet].

[5] Nielsen VML, Bruun NH, Søvsø MB, Kløjgård TA, Lossius HM, Bender L (2022). Pediatric emergencies in Helicopter Emergency Medical Services: a national population-based cohort study from Denmark. Ann Emerg Med.

[6] Enomoto Y, Yusuke TMD, Takahiro K, Kasuki N, Asuka T (2024). Association between helicopter medical services for pediatric trauma patients and mortality: systematic review and meta-analysis. Am J Emerg Med.

[7] Loyd JW, Larsen T, Kuhl EA, Swanson D (2025). Aeromedical transport. In: StatPearls [Internet].

[8] Berkowitz D, Cohen JS, McCollum N, Rojas CR, Chamberlain JM (2023). Delays in treatment and disposition attributable to undertriage of pediatric emergency medicine patients. Am J Emerg Med.

[9] Scaife JH, Bryce JR, Iantorno SE, Yang M, McCrum ML, Bucher BT (2024). Secondary undertriage of pediatric trauma patients across United States emergency departments. J Surg Res.

[10] Tsuchiya EA, Rosenkrantz O, Hansen TM, Petersen JAK, Steinmetz J (2025). Prehospital triage of pediatric emergencies treated by Helicopter Emergency Medical Services: a population-based cohort study. Air Med J.

[11] Brasil. Ministério da Saúde. Agência Nacional de Vigilância Sanitária. (2017). Assistência Segura: uma reflexão teórica aplicada à prática [Internet].

[12] Freitas L, Lacerda A (2025). The role of aviation in emergency medical care: a narrative review on aeromedical transport and prehospital rescue. Braz J Health Rev.

[13] Sociedade Brasileira de Pediatria (2024). Diretrizes para o transporte aeromédico em paciente pediátrico/neonatal em aeronave de asa rotativa [Internet].

[14] Batista EA (2022). Protocolo de cuidados com a fixação para o transvoo do paciente pediátrico após o resgate aeromédico em aeronave de asa rotativa [dissertação].

[15] Araiza A, Duran M, Surani S, Varon J (2021). Aeromedical Transport of Critically Ill Patients: A Literature Review. Cureus.

[16] Cochran-Caggiano N, Till S, Holt C, Lang N, Ata A, Cerone J (2023). Children and restraints study in emergency ambulance transport: an observational study and analysis of current pediatric ambulance transport practices. Pediatr Emerg Care.

[17] Pimenta CAM (2015). Conselho Regional de Enfermagem de São Paulo. Guia para construção de protocolos assistenciais de enfermagem [Internet].

[18] Mortamet G, Harrington K, Raffin H, Menat Y, Oualha M, Renolleau S (2020). Aeromedical transport in children: a descriptive analysis of 96 cases. Pediatr Emerg Care.

[19] Matos RI, Jenkins-Woodrum K, Clemons MA (2026). The experience of critical care air transport teams that transported Afghan refugee children: a qualitative thematic analysis. Mil Med.

[20] Wilson MA, Cutcliffe JR, Armitage CNH, Eaton KN (2020). Moral distress in the critical care air transport nurse. Nurs Outlook.

[21] Ryu MY, Martin MJ, Jin AH, Tabor HK, Wren SM (2023). Characterizing Moral Injury and Distress in US Military Surgeons Deployed to Far-Forward Combat Environments in Afghanistan and Iraq. JAMA Netw Open.

