# Supplementary material for: Pediatric patient fixation for air aeromedical transport in rotary-wing aircraft: a professional experience report
Source: Rev Esc Enferm USP. 2026 Jul 24;60:e20260017. doi: 10.1590/1980-220X-REEUSP-2026-0017en (PMC13419339; doi:10.1590/1980-220X-REEUSP-2026-0017en)
Supplement: Translation

# Translation: Fixação do paciente pediátrico no transporte aeromédico em aeronave de asa rotativa: relato de experiência profissional

**DOI:** 10.1590/1980-220X-REEUSP-2026-0017pt

**Authors:** Euseli de Assis Batista, José Colleti

**Affiliations:** 1 Hospital lsraelita Albert Einstein, São Paulo, SP, Brasil.

**Keywords:** Aeronave, Resgate Aéreo, Emergências, Pediatría, Guias como Assunto.

## Abstract

**Objetivo:**

Relatar a experiência de elaboração de um protocolo de cuidados para a fixação do paciente pediátrico durante o transvoo em aeronave de asa rotativa.

**Método:**

Relato de experiência profissional. O protocolo foi elaborado no serviço aeromédico de urgência e emergência de Santa Catarina, orientado pelo Guia para a Construção de Protocolos Assistenciais do Coren/São Paulo. A fundamentação teórica baseou-se em revisão integrativa e consulta a documentos de órgãos oficiais nacionais e internacionais, bem como do Conselho Federal de Enfermagem. Realizou-se validação interna com oito enfermeiros de voo do serviço.

**Resultados:**

O protocolo estruturou os cuidados de fixação do paciente pediátrico em três momentos assistenciais: pré-voo, transvoo e pós-voo, apoiando a tomada de decisão clínica e a segurança durante o transporte.

**Considerações Finais:**

O protocolo desenvolvido padronizou as práticas de fixação do paciente pediátrico no serviço aeromédico, promovendo a segurança e o suporte à tomada de decisão clínica durante o transvoo, minimizando o risco de agravos durante o transporte e fortalecendo a prática profissional no âmbito do atendimento aeromédico.

## INTRODUÇÃO

O transporte aeromédico consiste no resgate ou remoção de indivíduos acometidos por situações graves de saúde, realizado por meio de aeronaves de asa fixa ou rotativa, integradas ao sistema de atendimento pré-hospitalar e à central de regulação médica de urgências^(1)^.

Os serviços aeromédicos de emergência por aeronave de asa rotativa são norteados pelas recomendações internacionais da *Air Medical Physicians Association*, a *National Association of EMS Physicians* e o *American College of Surgeons Committee on Trauma* ^(2)^. No Brasil, o serviço aeromédico é normatizado pela Portaria nº 2048/2002 do Ministério da Saúde para o exercício da atividade profissional em ambulância tipo E, compreendidas pelas aeronaves de asa fixa e asa rotativa regulamentadas de acordo com as normas da Agência Nacional de Aviação Civil^(3)^. Em relação aos profissionais que atuam no serviço aeromédico, o enfermeiro é o responsável pela assistência e pelo gerenciamento do atendimento pré-hospitalar móvel e inter-hospitalar em veículo aéreo, sendo normatizado pela Resolução nº 660/2021 do Conselho Federal de Enfermagem^(4)^.

O transporte aeromédico por aeronave de asa rotativa abrange todas as faixas etárias, presta atendimento rápido a pacientes em condições agudas potencialmente fatais, emergências cardíacas, neurológicas ou vasculares, trauma grave e lesões acidentais^(5,6,7)^.

Esta modalidade de transporte, realizado para pacientes pediátricos, tem como indicação aqueles gravemente enfermos ou feridos, geralmente identificados pela equipe do serviço de aeromédico no período pré-hospitalar, resultando na triagem para os serviços de emergência terciária^(8,9)^. No contexto pediátrico, as particularidades anatômicas e fisiológicas do paciente pediátrico potencializam a vulnerabilidade ao trauma, como a desproporção entre cabeça e corpo na primeira infância, que eleva a frequência de quedas e o risco de traumatismos, inclusive craniano^(10)^. Ainda, a menor massa corporal e a proximidade entre os órgãos favorecem maior dissipação de energia nos impactos pela flexibilidade esquelética, ampliando a probabilidade de lesões sistêmicas e favorecendo a ocorrência de lesões viscerais sem sinais externos evidentes^(10)^.

Ademais, os pacientes pediátricos são mais vulneráveis à hipotermia quando acometidos por trauma^(10)^. Em relação às vias aéreas, apresentam a língua relativamente maior, cavidade oral menor, aumentando o risco de obstrução da via aérea por corpos estranhos (engasgamento) pelo comportamento exploratório até os três anos, ou mesmo obstrução pelo relaxamento da língua nos casos de inconsciência^(10)^.

Em vista disso, a avaliação clínica de pacientes pediátricos representa um desafio significativo para a equipe do aeromédico. Um estudo de coorte dinamarquês relatou que 6,4% das emergências pediátricas atendidas pelo serviço, inicialmente avaliadas como “não críticas”, necessitam https://www-sciencedirect-com.ez46.periodicos.capes.gov.br/science/article/pii/S1067991X25001579?via%3Dihub#bib0007de cuidados intensivos nas 24 horas https://www-sciencedirect-com.ez46.periodicos.capes.gov.br/science/article/pii/S1067991X25001579?via%3Dihub#bib0008seguintes^(5)^. Diante do exposto, cabe destacar que o transporte aeromédico é fundamental no manejo de situações de urgência e emergência, especialmente em condições com barreiras geográficas ou longas distâncias da ocorrência^(11)^.

No entanto, esse tipo de transporte impõe desafios operacionais e assistenciais, em virtude das condições ambientais de voo, espaço da aeronave, vibrações, ruídos e adaptação para a realização dos procedimentos clínicos em ambiente aéreo^(12)^. Logo, as equipes que atendem/transportam crianças devem estar preparadas com todo o espectro de tamanhos de equipamentos necessários para a fixação de um paciente pediátrico que compreende desde o prematuro (< 500 gramas) até uma criança com 100 kg ou mais^(13)^.

Nesse sentido, a partir da prática profissional enquanto enfermeira de voo do transporte aeromédico, identificou-se uma lacuna relacionada à ausência de padronização da fixação segura do paciente pediátrico em aeronave de asa rotativa. A revisão de literatura realizada confirmou a escassez de protocolos específicos voltados a essa finalidade^(14)^, corroborada pela ausência de diretrizes internacionais que regulem o padrão de cuidado durante o transporte aeromédico^(15)^ e pela variabilidade das práticas de contenção pediátrica identificada na literatura^(16)^. Este estudo tem como objetivo relatar a experiência de elaboração de um protocolo de cuidados para a fixação do paciente pediátrico durante o transvoo em aeronave de asa rotativa.

## MÉTODO

### Tipo do Estudo

Trata-se de um estudo do tipo relato de experiência profissional, o qual descreve a elaboração de um protocolo assistencial de enfermagem voltado para a fixação de pacientes pediátricos durante o transporte aeromédico em aeronave de asa rotativa.

### Local

O protocolo foi desenvolvido e implementado no serviço aeromédico de urgência e emergência, no contexto do Serviço Móvel de Urgência (SAMU) de Santa Catarina, onde é denominado de “Arcanjos”. O Arcanjo opera com dois helicópteros – asa rotativa modelo esquilo arcanjo 01 com base em Florianópolis e a outra aeronave arcanjo 03 com base no aeroporto de Blumenau. Os arcanjos de asa rotativa apresentam diferentes configurações, quanto à tripulação e ao tipo de operação, e podem ser classificados como de alto risco ou alta complexidade. Com base na ocorrência, a tripulação da aeronave pode compor: dois pilotos (duplo comando), tripulante operacional, médico e enfermeiro de voo. Em operações de alto risco ou de alta complexidade, poderá ser embarcado o sexto tripulante operacional resgatista.

### População

O protocolo elaborado abrange pacientes pediátricos, atendidos pelo serviço aeromédico de urgência e emergência.

### Coleta de Dados

A elaboração do protocolo foi orientada pelo Guia para a Construção de Protocolos Assistenciais do Conselho Regional de Enfermagem de São Paulo^(17)^. Esse guia orienta a construção de protocolos em etapas sequenciais, compreendendo: definição do tema e escopo do protocolo, levantamento bibliográfico e revisão das evidências científicas, elaboração do conteúdo e fluxogramas, validação interna com os profissionais envolvidos e implementação no serviço.

Para a fundamentação teórica, realizou-se uma revisão integrativa de literatura, nas bases de dados *Medical Literature Analysis and Retrieval System Online*, *Cumulative Index to Nursing and Allied*, Scopus, Literatura Latino-Americana e do Caribe em Ciências da Saúde, Embase; Base de Dados de Enfermagem e *Scientific Electronic Library Online*, onde utilizaram-se os Descritores de Ciências da Saúde e *Medical Subject Headings*: *“Ambulância aérea”, “Serviço aeromédico”, “Paciente pediátrico”, “Helitransportados”, “Helicóptero de emergência”, “Ambulância aérea”, “Resgate aéreo”, “Atendimento de emergência pré-hospitalar”.* A busca compreendeu o período de 2010 a 2022, resultando inicialmente em 412 artigos. Após a aplicação dos critérios de inclusão e exclusão, leitura de títulos e resumos e texto na íntegra, a amostra foi composta por quatro estudos que abordavam sobre o transporte aeromédico em pacientes pediátricos e aspectos relacionados à segurança.

Complementarmente, foram consultados documentos normativos do Ministério da Saúde e órgãos oficiais nacionais e internacionais, bem como Resoluções do Conselho Federal de Enfermagem, com o objetivo de incorporar recomendações legais e técnico-assistenciais que orientam a prática profissional em serviços aeromédicos. Embasado nas evidências científicas e normativas identificadas, o protocolo foi estruturado contemplando cuidados de fixação do paciente pediátrico em três momentos: cuidados pré-voo, cuidados no transvoo relacionados à fixação do paciente pediátrico e cuidados no pós-voo.

A etapa de validação interna do protocolo foi realizada junto a oito enfermeiros de voo atuantes no serviço aeromédico de urgência e emergência de Santa Catarina. Após a validação interna, o protocolo foi implementado no serviço aeromédico como instrumento orientador da prática assistencial, sendo utilizado no transporte aeromédico de pacientes pediátricos, com o objetivo de padronizar as condutas de fixação e apoiar a tomada de decisão clínica durante o atendimento. O presente relato de experiência detalha a construção do protocolo especificamente, não detalhando as etapas de validação interna e implementação no serviço aeromédico de urgência e emergência.

### Aspectos Éticos

Por se tratar de um relato de experiência profissional, não houve necessidade de submissão ao Comitê de Ética em Pesquisa (CEP), em conformidade com a Resolução CNS nº 510/2016.

## RESULTADOS

A experiência resultou na elaboração de um protocolo assistencial voltado para a fixação segura do paciente pediátrico durante o transvoo em aeronave de asa rotativa. Este foi estruturado a partir de evidências científicas, normativas nacionais e internacionais e da prática assistencial do serviço aeromédico, sendo validado internamente por enfermeiros de voo.

O protocolo estruturou os cuidados de fixação do paciente pediátrico em três momentos assistenciais: pré-voo, transvoo e pós-voo. Essa organização possibilita sistematizar condutas, reduzir a variabilidade da prática clínica e apoiar a tomada de decisão da equipe em cenário de alta complexidade.

### Cuidados No Pré-Voo

Os cuidados pré-voo compreendem a explanação das atribuições dos profissionais enfermeiros e médicos, bem como modelos de aeronaves e a composição das tripulações.

Compete ao enfermeiro a checagem do funcionamento dos equipamentos, da disponibilidade e validade de materiais, bem como a reposição dos insumos utilizados durante o atendimento, assegurando a prontidão assistencial para o transporte aeromédico pediátrico. Cabe ainda participar, juntamente com o médico, da tomada de decisão quanto ao transporte, realizar o preenchimento e a evolução dos pacientes atendidos e, ao final do atendimento, proceder à limpeza dos equipamentos e ao encaminhamento dos materiais para desinfecção^(3,4)^.

À equipe médica compete a checagem do funcionamento dos equipamentos e da disponibilidade dos materiais na mochila, a reposição dos insumos utilizados, o preenchimento das evoluções dos pacientes após cada ocorrência e a prescrição das medicações controladas administradas durante o atendimento^(4)^.

Quanto aos modelos das aeronaves, incluem-se o Modelo Esquilo (HB – 350 B1), prefixo PT, denominado “Helicóptero Arcanjo 01”’ com capacidade para transportar seis pessoas, sendo dois pilotos, um tripulante operacional, um médico, um enfermeiro e um paciente, não dispondo de kit aeromédico. O Modelo Esquilo (AS- 350 B2), prefixo PR- BNU, denominado “Helicóptero Arcanjo 03”, possui capacidade semelhante, com a diferença de contar com o kit aeromédico disponível para o atendimento.

### Cuidados No Transvoo Relacionados À Fixação do Paciente Pediátrico

Durante o acionamento, são obtidas informações referentes à história clínica, idade e possíveis patologias da criança. A equipe realiza a checagem da disponibilidade dos equipamentos para realização de manobras de extração da vítima, testa o funcionamento de cada aparelho e prepara a aeronave com materiais e equipamentos, organizando-os de forma a não comprometer a assistência durante o transvoo. Em conjunto com o médico, procede-se à organização dos equipamentos, materiais e medicações, estabelecendo uma disposição que favoreça o acesso rápido e seguro, proporcionando uma remoção segura da criança^(3)^.

 Em relação aos equipamentos disponíveis, o serviço conta com a maca rígida de transporte e *Kendrick Extrication Device* (KED) adulto e pediátrico. A configuração de peso e sugestão de uso padronizadas visam o conforto da criança a ser transportada. Ressalta-se que há crianças cujas estaturas não condizem com a idade cronológica, sendo avaliada, no momento do atendimento, a adequação do tamanho do equipamento utilizado^(4)^. A maca rígida utilizada como dispositivo de apoio ao transporte apresenta capacidade de suporte até 180 kg e possibilita a fixação segura do paciente pediátrico, além de permitir a realização de exames e procedimentos sem necessidade de remoção imediata do dispositivo. Dispõe de cintos de segurança nas cores preto, vermelho, verde e amarelo^(4)^.

O KED adulto ou pediátrico consiste em um colete de imobilização dorsal associado à prancha rígida, sendo indicado para vítimas de traumas que se encontram sentadas, configurando-se como uma opção no transporte de crianças no serviço pré-hospitalar. A prancha rígida de imobilização é dimensionada para o transporte de pessoas com até 180 kg. Ambos dispositivos possuem estrutura radiotransparente, alças em nylon com engates rápidos, pegadores manuais, faixa para proteção do queixo e testa, almofadas e tirantes de engate rápidos nas cores padrão (amarelo, verde, vermelho), permitindo sua utilização durante a realização de exames quando necessários^(4)^ (Figura 1).

**Figura 1:** Equipamentos para o transporte. Florianópolis, SC, Brasil, 2025.

Ao realizar a imobilização da criança para o transvoo no ked de tamanho apropriado, o uso de manta térmica é considerado indispensável para controle de temperatura corporal. A sequência da fixação prioriza inicialmente a cabeça, seguida do tronco e dos membros. Na posição anatômica, os espaços vazios são preenchidos com coxins, ou por meio da elevação do tronco da criança quando indicado. Destaca-se que a fixação deve ser realizada de modo a garantir estabilidade e segurança, sem comprometer os movimentos respiratórios (Figura 2).

**Figura 2:** Fixação do paciente pediátrico com Kendrick Extrication Device. Florianópolis, SC, Brasil, 2025.

O apoio para o deslocamento com a maca rígida finaliza a sequência da segurança no transvoo, considerando as lesões e/ou a condição clínica apresentada, permitindo assistência adequada durante o transporte. O equipamento dispõe de cintas de fixação do tipo tira-aranhas, contribuindo para maior estabilidade da criança durante o voo.

A criança não deve ser transportada no colo de um adulto durante o transvoo, em virtude dos riscos de queda ou trauma, especialmente em situações de turbulência ou manobras da aeronave. A fixação da criança no banco da aeronave possibilita melhor observação clínica por parte da equipe, incluindo a avaliação da tonalidade da pele, ocorrência de crise convulsiva, êmese ou alterações comportamentais, além de viabilizar a realização de compressões torácicas até a necessidade de pouso de emergência.

A decisão de interromper a imobilização, quando necessária para a segurança do paciente, deve ser devidamente documentada, com descrição detalhada do motivo. Ressalta-se que a imobilização não deve impedir a ventilação, a abertura das vias aéreas ou a realização de manobras de reanimação. Em situações específicas, pode ser mais adequado transportar a criança em sua própria cadeirinha de contenção veicular, quando esta se mostra mais segura. Crianças que reagem intensamente às tentativas de imobilização podem apresentar maior risco de agravamento de lesões vertebrais; nesses casos, estratégias alternativas são consideradas, como distração ou orientação para permanência imóvel, sem contenção rígida^(3,10)^.

Antes do transvoo, as prioridades de cuidado do paciente devem ser avaliadas e sistematizadas, incluindo a revisão de todos os equipamentos necessários, a realização de uma fixação segura e o registro de enfermagem de forma objetiva e clara de quaisquer intercorrências ocorridas durante o pré-voo^(3,4)^. Diante dos riscos inerentes ao transvoo, os cuidados relacionados à fixação do paciente pediátrico são essenciais para evitar o agravamento do quadro clínico. A fixação da criança ao banco da aeronave favorece a segurança do paciente e possibilita à equipe a identificação precoce de alterações clínicas, como crise convulsiva, êmese ou mudança na coloração da pele. A associação dos tirantes do ked, das tiras-aranhas da maca rígida e dos cintos da aeronave proporciona maior estabilidade, minimizando movimentos bruscos durante o transvoo e contribuindo para a integridade física e psíquica do paciente pediátrico^(4)^.

Durante o transvoo, o médico e o enfermeiro posicionam-se ajoelhados no assoalho da aeronave em razão do espaço físico reduzido e da limitação de mobilidade. Dessa forma, procedimentos de suporte avançado de vida, como intubação orotraqueal, compressões torácicas, desfibrilação cardíaca e drenagem torácica, são previstos previamente ao embarque.

O protocolo estabelece que o transvoo do paciente pediátrico sem ventilação mecânica deve ser realizado pela porta esquerda da aeronave, com entrada na posição cefálica até a completa acomodação caudal. A escolha pela porta esquerda nessa situação justifica-se pela configuração interna da aeronave, que favorece o posicionamento da cabeceira do paciente voltada para a equipe assistencial, permitindo melhor acesso às vias aéreas e à monitorização durante o voo. Após a entrada, procede-se à fixação dos cintos de segurança e à ancoragem da prancha rígida, juntamente com os equipamentos necessários, conforme a idade e o peso da criança. Em crianças com peso aproximado de 7 kg e idade inferior a 1 ano, o protocolo preconiza o uso do KED pediátrico. Para crianças com idade superior a um ano e peso acima de 7 kg, o protocolo indica o uso do dispositivo adulto, associado à prancha rígida para o deslocamento até a aeronave^(4)^.

Para os pacientes pediátricos em ventilação mecânica, o protocolo orienta que a entrada no transvoo ocorra pela porta direita da aeronave, da posição caudal até a completa entrada cefálica. Essa conduta justifica-se pela necessidade de posicionar o paciente com a extremidade cefálica próxima aos equipamentos de ventilação, facilitando o acesso e o controle dos dispositivos durante o voo. Cabe destacar que a escolha da porta de acesso pode requerer adaptações conforme o local de pouso e as condições do terreno, sendo necessário avaliar, em cada situação, se o paciente deverá ser introduzido na aeronave com a cabeça ou os pés na direção da entrada. Após a acomodação, realiza-se a fixação dos cintos de segurança e a ancoragem da prancha rígida no banco da tripulação, bem como a instalação dos equipamentos de monitorização. Nesses casos, o uso do KED, adulto ou pediátrico, associado à prancha rígida, favorece a segurança no deslocamento e o suporte para acomodação dos equipamentos de ventilação e monitorização, reforçando a estabilidade do paciente durante o transvoo^(4)^.

### Cuidados No Pós-Voo

No pós-voo, são registradas e repassadas, tanto pelo médico quanto pelo enfermeiro, todas as informações e intercorrências ocorridas antes, durante e após a chegada da criança ao destino de tratamento. Procede-se à reposição dos insumos e equipamentos utilizados durante a remoção do paciente pediátrico, bem como à limpeza e à desinfecção do interior da aeronave e dos dispositivos de fixação empregados no transvoo, assegurando a prontidão do serviço para novos acionamentos^(3,4)^.

No protocolo elaborado consta, ainda, um fluxograma assistencial para o atendimento do paciente pediátrico, com a descrição das etapas correspondentes a cada fase do cuidado durante o transporte aeromédico (Figura 3).

**Figura 3:** Fluxograma de acionamento para atendimento do paciente pediátrico. Florianópolis, SC, Brasil, 2025.

## DISCUSSÃO

O transporte aeromédico de pacientes pediátricos em aeronave de asa rotativa configura-se como uma prática de alta complexidade assistencial, na qual a segurança do paciente depende diretamente da adequação dos dispositivos de fixação e da organização da assistência prestada durante todas as etapas do voo. Estudos internacionais evidenciam que a variabilidade nas práticas de imobilização e fixação durante o transporte aéreo pode aumentar o risco de eventos adversos, especialmente em pacientes pediátricos com condições clínicas instáveis ou em ventilação mecânica^(16,18)^.

Nesse contexto, a experiência relatada demonstra que a elaboração de um protocolo assistencial específico para a fixação do paciente pediátrico contribuiu para a padronização das condutas e para a redução da variabilidade da prática clínica no serviço aeromédico. A organização dos cuidados ocorreu em três momentos: pré-voo, transvoo e pós-voo, que favoreceu o planejamento antecipado, a mitigação de riscos e a continuidade do cuidado, aspectos considerados essenciais em cenários caracterizados por restrições físicas, vibração, ruído e limitação de acesso ao paciente durante o voo^(2,7)^.

No pré-voo, a definição de critérios para checagem de equipamentos e seleção de dispositivos de fixação, conforme idade, peso, estatura e condição clínica da criança, mostrou-se fundamental para a segurança do transporte. A literatura aponta que a inadequação do dispositivo de contenção ao perfil do paciente pediátrico está associada a maior instabilidade durante o deslocamento e dificuldade na realização de intervenções emergenciais^(19)^. Assim, a sistematização dessa etapa contribui para a antecipação de demandas assistenciais e para a tomada de decisão clínica mais assertiva.

Durante o transvoo, a padronização da aplicação dos dispositivos de fixação, priorizando a estabilidade, a manutenção das vias aéreas e a possibilidade de monitorização contínua, encontra respaldo nas recomendações internacionais para o transporte aeromédico pediátrico. As especificidades anatômicas e fisiológicas dessa população, com maior proporção cefálica, flexibilidade do esqueleto e maior vulnerabilidade a hipotermia, exigem estratégias de imobilização que garantam a segurança sem comprometer a ventilação ou o acesso às vias aéreas^(10)^.

O uso do KED associado a prancha rígida, conforme descrito no protocolo, mostrou-se compatível com essas recomendações, ao permitir estabilidade adequada e suporte às intervenções necessárias durante o voo. Outro aspecto relevante refere-se à possibilidade de utilização de dispositivos alternativos de contenção, como cadeirinha veicular, em situações específicas. Evidências apontam que, quando adequadamente fixados, esses dispositivos podem representar uma alternativa segura no transporte aeromédico, especialmente em crianças que apresentam intensa reação a imobilidade rígida^(16)^.

No pós-voo, a padronização do registro das informações assistenciais e da reorganização operacional do serviço contribui para a continuidade do cuidado e para a segurança do paciente, além de favorecer a rastreabilidade das condutas adotadas. A literatura destaca que registros sistematizados são essenciais para a comunicação entre equipes e para a avaliação da qualidade da assistência em serviços de urgência e emergência^(3)^.

Destaca-se, ainda, o papel do enfermeiro de voo como profissional central na organização da assistência e na gestão dos recursos durante o transporte aeromédico. A atuação do enfermeiro na checagem de equipamentos, na aplicação dos dispositivos de fixação e na tomada de decisão compartilhada com a equipe médica está em consonância com as normativas do Conselho Federal de Enfermagem, que reconhecem sua responsabilidade tanto assistencial quanto gerencial no atendimento pré-hospitalar e inter-hospitalar em veículo aéreo^(4)^. Estudos apontam a importância do preparo dos profissionais de saúde, pois realizar procedimentos que salvam vidas de crianças sem as ferramentas ou o treinamento adequado representa um desafio significativo para a equipe^(20,21)^.

Diante do exposto, o protocolo contribui para a padronização da fixação do paciente pediátrico durante o transvoo, fortalecendo a autonomia profissional e subsidiando a tomada de decisão clínica em cenário de alta complexidade, no qual o tempo e o espaço impõem limitações importantes à assistência. Como limitação do estudo, destaca-se a escassez de publicações científicas voltadas especificamente para a prática da enfermagem na fixação do paciente pediátrico durante o transvoo aeromédico.

Como contribuição para a área da saúde, o protocolo elaborado descreve os principais cuidados de fixação durante o transvoo de pacientes pediátricos, reunindo informações essenciais para a segurança do paciente e apresentando potencial de aplicabilidade em outros contextos aeromédicos.

## CONCLUSÃO

O transporte aeromédico de pacientes pediátricos em aeronave de asa rotativa expõe os pacientes a riscos inerentes que podem interferir significativamente em seu estado de saúde, exigindo medidas preventivas e terapêuticas sistematizadas para minimizar as complicações durante o deslocamento. A experiência relatada evidencia que a elaboração de um protocolo de cuidados voltado para a fixação do paciente pediátrico contribui para qualificar a assistência, ao sistematizar condutas e reduzir a variabilidade do cuidado em um contexto de alta complexidade. O protocolo desenvolvido padronizou as práticas de fixação do paciente pediátrico no serviço aeromédico Arcanjo, promovendo a padronização, a segurança e o suporte à tomada de decisão clínica durante o transvoo, reduzindo o risco de agravos secundários durante o transporte e fortalecendo a prática profissional no âmbito do atendimento aeromédico.

## Data Availability

Conforme preconizado pela Ciência Aberta, o link do repositório no qual os dados de pesquisa foram depositados: https://repositorio.ufsc.br/xmlui/bitstream/handle/123456789/247267/PGCF0174-D.pdf?sequence=1.

# Translation: Inmovilización del paciente pediátrico en el transporte aeromédico en aeronaves de ala giratoria: informe de experiencia profesional

**DOI:** 10.1590/1980-220X-REEUSP-2026-0017es

**Authors:** Euseli de Assis Batista, José Colleti

**Affiliations:** 1 Hospital lsraelita Albert Einstein, São Paulo, SP, Brasil.

**Keywords:** Aeronaves, Ambulancias Aéreas, Urgencias médicas, Pediatría, Guías como Asunto.

## Abstract

**Objetivo:**

Relatar la experiencia de elaboración de un protocolo de cuidados para la inmovilización del paciente pediátrico durante el vuelo en aeronaves de ala giratoria.

**Método:**

Relato de experiencia profesional. El protocolo fue elaborado en el servicio aeromédico de urgencias y emergencias de Santa Catarina, orientado por la Guía para la Elaboración de Protocolos de Atención de Coren/São Paulo. La fundamentación teórica se basó en una revisión integrativa y en la consulta de documentos de organismos oficiales nacionales e internacionales, así como del Consejo Federal de Enfermería. Se llevó a cabo una validación interna con ocho enfermeros de vuelo del servicio.

**Resultados:**

El protocolo estructuró los cuidados de inmovilización del paciente pediátrico en tres momentos asistenciales: pre-vuelo, durante el vuelo y post-vuelo, apoyando la toma de decisiones clínicas y la seguridad durante el transporte.

**Consideraciones Finales:**

El protocolo desarrollado estandarizó las prácticas de inmovilización del paciente pediátrico en el servicio aeromédico, promoviendo la seguridad y el apoyo a la toma de decisiones clínicas durante el vuelo, minimizando el riesgo de complicaciones durante el transporte y fortaleciendo la práctica profesional en el ámbito de la atención aeromédica.

## INTRODUCCIÓN

El transporte aeromédico consiste en el rescate o traslado de personas afectadas por situaciones graves de salud, realizado mediante aeronaves de ala fija o rotatoria, integradas al sistema de atención prehospitalaria y a la central de regulación médica de urgencias^(1)^.

Los servicios aeromédicos de emergencia en aeronaves de ala giratoria se rigen por las recomendaciones internacionales de la *Air Medical Physicians Association*, la *National Association of EMS Physicians* y el *American College of Surgeons Committee on Trauma* ^(2)^. En Brasil, el servicio aeromédico está regulado por la Orden Ministerial n.º 2048/2002 del Ministerio de Salud para el ejercicio de la actividad profesional en ambulancias de tipo E, que comprenden las aeronaves de ala fija y de ala giratoria reguladas de acuerdo con las normas de la Agencia Nacional de Aviación Civil^(3)^. En cuanto a los profesionales que trabajan en el servicio aeromédico, el enfermero es el responsable de la asistencia y la gestión de la atención prehospitalaria móvil e interhospitalaria en vehículos aéreos, y está regulado por la Resolución n.º 660/2021 del Consejo Federal de Enfermería^(4)^.

El transporte aeromédico en aeronaves de ala giratoria abarca todos los grupos de edad y brinda atención rápida a pacientes en condiciones agudas potencialmente mortales, emergencias cardíacas, neurológicas o vasculares, traumatismos graves y lesiones accidentales^(5,6,7)^.

Esta modalidad de transporte, realizada para pacientes pediátricos, está indicada para aquellos gravemente enfermos o lesionados, generalmente identificados por el equipo del servicio aeromédico en el período prehospitalario, lo que da lugar a la derivación a los servicios de emergencia terciarios^(8,9)^. En el contexto pediátrico, las particularidades anatómicas y fisiológicas del paciente pediátrico aumentan la vulnerabilidad al trauma, como la desproporción entre la cabeza y el cuerpo en la primera infancia, lo que eleva la frecuencia de caídas y el riesgo de traumatismos, incluso craneales^(10)^. Además, la menor masa corporal y la proximidad entre los órganos favorecen una mayor disipación de energía en los impactos debido a la flexibilidad esquelética, lo que aumenta la probabilidad de lesiones sistémicas y favorece la aparición de lesiones viscerales sin signos externos evidentes^(10)^.

Asimismo, los pacientes pediátricos son más vulnerables a la hipotermia cuando sufren un traumatismo^(10)^. En cuanto a las vías respiratorias, presentan una lengua relativamente más grande y una cavidad oral más pequeña, lo que aumenta el riesgo de obstrucción de las vías respiratorias por cuerpos extraños (atragantamiento) debido al comportamiento exploratorio hasta los tres años, o incluso obstrucción por la relajación de la lengua en casos de inconsciencia^(10)^.

En vista de ello, la evaluación clínica de los pacientes pediátricos representa un desafío significativo para el equipo aeromédico. Un estudio de cohorte danés informó que el 6,4% de las emergencias pediátricas atendidas por el servicio, evaluadas inicialmente como “no críticas”, requieren cuidados intensivos en las 24 horas siguientes^(5)^. Ante lo expuesto, cabe destacar que el transporte aeromédico es fundamental en el manejo de situaciones de urgencia y emergencia, especialmente en condiciones con barreras geográficas o largas distancias desde el lugar del suceso^(11)^.

Sin embargo, este tipo de transporte plantea desafíos operativos y asistenciales, debido a las condiciones ambientales del vuelo, el espacio de la aeronave, las vibraciones, los ruidos y la adaptación para la realización de procedimientos clínicos en el entorno aéreo^(12)^. Por lo tanto, los equipos que atienden o transportan a niños deben estar preparados con toda la gama de tamaños de equipos necesarios para la inmovilización de un paciente pediátrico, que abarca desde el prematuro (< 500 gramos) hasta un niño de 100 kg o más^(13)^.

En este sentido, a partir de la práctica profesional como enfermera de vuelo en el transporte aeromédico, se identificó una laguna relacionada con la falta de estandarización de la sujeción segura del paciente pediátrico en aeronaves de ala giratoria.

La revisión bibliográfica realizada confirmó la escasez de protocolos específicos orientados a este fin^(14)^, corroborada por la ausencia de directrices internacionales que regulen el estándar de atención durante el transporte aeromédico^(15)^ y por la variabilidad de las prácticas de sujeción pediátrica identificadas en la literatura^(16)^. El objetivo de este estudio es relatar la experiencia de elaboración de un protocolo de cuidados para la sujeción del paciente pediátrico durante el vuelo en aeronaves de ala giratoria.

## MÉTODO

### Tipo de Estudio

Se trata de un estudio de tipo informe de experiencia profesional, que describe la elaboración de un protocolo de atención de enfermería orientado a la inmovilización de pacientes pediátricos durante el transporte aeromédico en aeronaves de ala giratoria.

### Lugar

El protocolo se desarrolló e implementó en el servicio aeromédico de urgencias y emergencias, en el marco del Servicio Móvil de Urgencias (SAMU) de Santa Catarina, donde se le denomina “Arcanjos”. El Arcanjo opera con dos helicópteros: el modelo Esquilo Arcanjo 01, con base en Florianópolis, y la otra aeronave, el Arcanjo 03, con base en el aeropuerto de Blumenau. Los Arcanjos de ala giratoria presentan diferentes configuraciones en cuanto a la tripulación y el tipo de operación, y pueden clasificarse como de alto riesgo o alta complejidad. Según la situación, la tripulación de la aeronave puede estar compuesta por: dos pilotos (doble mando), un tripulante operativo, un médico y un enfermero de vuelo. En operaciones de alto riesgo o alta complejidad, se puede incorporar un sexto tripulante operativo, que es un rescatista.

### Población

El protocolo elaborado abarca a pacientes pediátricos atendidos por el servicio aeromédico de urgencias y emergencias.

### Recolección de Datos

La elaboración del protocolo se basó en la Guía para la Elaboración de Protocolos de Atención del Consejo Regional de Enfermería de São Paulo^(17)^. Esta guía orienta la elaboración de protocolos en etapas secuenciales, que comprenden: definición del tema y el alcance del protocolo, revisión bibliográfica y de la evidencia científica, elaboración del contenido y los diagramas de flujo, validación interna con los profesionales involucrados e implementación en el servicio.

Para la fundamentación teórica, se realizó una revisión integrativa de la literatura en las bases de datos *Medical Literature Analysis and Retrieval System Online*, *Cumulative Index to Nursing and Allied*, Scopus, Literatura Latinoamericana y del Caribe en Ciencias de la Salud, Embase; Base de Datos de Enfermería y *Scientific Electronic Library Online*, donde se utilizaron los Descriptores de Ciencias de la Salud y *Medical Subject Headings*: *“Ambulância aérea”, “Serviço aeromédico”, “Paciente pediátrico”, “Helitransportados”, “Helicóptero de emergência”, “Ambulância aérea”, “Resgate aéreo”, “Atendimento de emergência pré-hospitalar”(“Ambulancia aérea”, “Servicio aeromédico”, “Paciente pediátrico”, “Transportados en helicóptero”, “Helicóptero de emergencia”, “Ambulancia aérea”, “Rescate aéreo”, “Atención de emergencia prehospitalaria”).* La búsqueda abarcó el período de 2010 a 2022, lo que dio como resultado inicialmente 412 artículos. Tras la aplicación de los criterios de inclusión y exclusión, la lectura de títulos y resúmenes y del texto completo, la muestra quedó compuesta por cuatro estudios que abordaban el transporte aeromédico en pacientes pediátricos y aspectos relacionados con la seguridad.

De manera complementaria, se consultaron documentos normativos del Ministerio de Salud y de organismos oficiales nacionales e internacionales, así como Resoluciones del Consejo Federal de Enfermería, con el objetivo de incorporar recomendaciones legales y técnico-asistenciales que orientan la práctica profesional en los servicios aeromédicos. Basado en las evidencias científicas y normativas identificadas, el protocolo se estructuró contemplando los cuidados de inmovilización del paciente pediátrico en tres momentos: cuidados previos al vuelo, cuidados durante el vuelo relacionados con la inmovilización del paciente pediátrico y cuidados posteriores al vuelo.

La etapa de validación interna del protocolo se llevó a cabo con ocho enfermeros de vuelo que trabajan en el servicio aeromédico de urgencias y emergencias de Santa Catarina. Tras la validación interna, el protocolo se implementó en el servicio aeromédico como instrumento orientador de la práctica asistencial, utilizándose en el transporte aeromédico de pacientes pediátricos, con el objetivo de estandarizar los procedimientos de inmovilización y apoyar la toma de decisiones clínicas durante la atención. El presente informe de experiencia detalla específicamente la elaboración del protocolo, sin detallar las etapas de validación interna e implementación en el servicio aeromédico de urgencias y emergencias.

### Aspectos Éticos

Al tratarse de un informe de experiencia profesional, no fue necesario someterlo al Comité de Ética en Investigación (CEP), de conformidad con la Resolución CNS n.º 510/2016.

## RESULTADOS

La experiencia dio lugar a la elaboración de un protocolo de atención orientado a la inmovilización segura del paciente pediátrico durante el vuelo en una aeronave de ala giratoria. Este se estructuró a partir de evidencia científica, normativas nacionales e internacionales y la práctica asistencial del servicio aeromédico, y fue validado internamente por enfermeros de vuelo.

El protocolo estructuró los cuidados de sujeción del paciente pediátrico en tres momentos asistenciales: pre-vuelo, durante el vuelo y post-vuelo. Esta organización permite sistematizar las conductas, reducir la variabilidad de la práctica clínica y apoyar la toma de decisiones del equipo en un escenario de alta complejidad.

### Cuidados Previos al Vuelo

Los cuidados previos al vuelo comprenden la explicación de las funciones de los profesionales de enfermería y médicos, así como los modelos de aeronaves y la composición de las tripulaciones.

Corresponde al enfermero verificar el funcionamiento de los equipos, la disponibilidad y validez de los materiales, así como la reposición de los insumos utilizados durante la atención, asegurando la prontitud asistencial para el transporte aeromédico pediátrico. También le corresponde participar, junto con el médico, en la toma de decisiones sobre el transporte, completar los registros y la evolución de los pacientes atendidos y, al finalizar la atención, proceder a la limpieza de los equipos y al envío de los materiales para su desinfección^(3,4)^.

Al equipo médico le corresponde verificar el funcionamiento de los equipos y la disponibilidad de los materiales en la mochila, reponer los insumos utilizados, completar los informes de evolución de los pacientes después de cada caso y prescribir los medicamentos controlados administrados durante la atención^(4)^.

En cuanto a los modelos de aeronaves, se incluye el Modelo Esquilo (HB – 350 B1), prefijo PT, denominado “Helicóptero Arcanjo 01”, con capacidad para transportar a seis personas: dos pilotos, un tripulante operativo, un médico, un enfermero y un paciente, sin disponer de kit aeromédico. El modelo Esquilo (AS-350 B2), con prefijo PR-BNU, denominado “Helicóptero Arcanjo 03”, tiene una capacidad similar, con la diferencia de que cuenta con un kit aeromédico disponible para la atención.

### Cuidados Durante el Vuelo Relacionados con la Sujeción del Paciente Pediátrico

Durante la activación, se recopila información sobre el historial clínico, la edad y las posibles patologías del niño. El equipo verifica la disponibilidad de los equipos para realizar las maniobras de extracción de la víctima, prueba el funcionamiento de cada aparato y prepara la aeronave con materiales y equipos, organizándolos de manera que no comprometa la asistencia durante el traslado. En colaboración con el médico, se procede a la organización de los equipos, materiales y medicamentos, estableciendo una disposición que favorezca el acceso rápido y seguro, lo que permite un traslado seguro del niño^(3)^.

En cuanto a los equipos disponibles, el servicio cuenta con la camilla rígida de transporte y el *Kendrick Extrication Device* (KED) para adultos y niños. La configuración de peso y las recomendaciones de uso estandarizadas tienen como objetivo la comodidad del niño que se va a transportar. Cabe destacar que hay niños cuya estatura no se corresponde con su edad cronológica, por lo que, en el momento de la atención, se evalúa la adecuación del tamaño del equipo utilizado^(4)^.

La camilla rígida utilizada como dispositivo de apoyo para el transporte tiene una capacidad de soporte de hasta 180 kg y permite la sujeción segura del paciente pediátrico, además de permitir la realización de exámenes y procedimientos sin necesidad de retirar inmediatamente el dispositivo. Cuenta con cinturones de seguridad en los colores negro, rojo, verde y amarillo^(4)^.

El KED para adultos o pediátrico consiste en un chaleco de inmovilización dorsal asociado a una tabla rígida, indicado para víctimas de traumatismos que se encuentran sentadas, lo que lo configura como una opción para el transporte de niños en el servicio prehospitalario. La tabla rígida de inmovilización está dimensionada para el transporte de personas de hasta 180 kg. Ambos dispositivos cuentan con una estructura radiotransparente, correas de nylon con enganches rápidos, asas manuales, banda para la protección de la barbilla y la frente, almohadillas y tirantes de enganche rápido en los colores estándar (amarillo, verde, rojo), lo que permite su uso durante la realización de exámenes cuando sea necesario^(4)^(Figura 1).

**Figura 1:** Equipos para el transporte. Florianópolis, SC, Brasil, 2025.

Al inmovilizar al niño para el traslado en una camilla de tamaño adecuado, se considera indispensable el uso de una manta térmica para controlar la temperatura corporal. La secuencia de sujeción prioriza inicialmente la cabeza, seguida del tronco y las extremidades. En la posición anatómica, los espacios vacíos se rellenan con almohadillas o mediante la elevación del tronco del niño cuando sea indicado. Cabe destacar que la sujeción debe realizarse de manera que se garantice la estabilidad y la seguridad, sin comprometer los movimientos respiratorios (Figura 2).

**Figura 2:** Inmovilización del paciente pediátrico con el Kendrick Extrication Device. Florianópolis, SC, Brasil, 2025.

El soporte para el traslado con la camilla rígida completa la secuencia de seguridad durante el vuelo, teniendo en cuenta las lesiones y/o el estado clínico del paciente, lo que permite una asistencia adecuada durante el transporte. El equipo cuenta con correas de sujeción tipo araña, lo que contribuye a una mayor estabilidad del niño durante el vuelo.

No se debe transportar al niño en brazos de un adulto durante el vuelo, debido al riesgo de caídas o traumatismos, especialmente en situaciones de turbulencia o maniobras de la aeronave. La sujeción del niño en el asiento de la aeronave permite una mejor observación clínica por parte del equipo, incluyendo la evaluación del tono de la piel, la aparición de crisis convulsivas, vómitos o alteraciones del comportamiento, además de permitir la realización de compresiones torácicas hasta que sea necesario realizar un aterrizaje de emergencia.

La decisión de interrumpir la inmovilización, cuando sea necesaria para la seguridad del paciente, debe documentarse debidamente, con una descripción detallada del motivo. Cabe destacar que la inmovilización no debe impedir la ventilación, la apertura de las vías respiratorias o la realización de maniobras de reanimación. En situaciones específicas, puede ser más adecuado transportar al niño en su propia silla de seguridad para vehículos, cuando esta resulte más segura. Los niños que reaccionan intensamente a los intentos de inmovilización pueden presentar un mayor riesgo de agravamiento de las lesiones vertebrales; en estos casos, se consideran estrategias alternativas, como la distracción o la orientación para permanecer inmóvil, sin sujeción rígida^(3,10)^.

Antes del vuelo, deben evaluarse y sistematizarse las prioridades de atención al paciente, incluyendo la revisión de todo el equipo necesario, la realización de una sujeción segura y el registro de enfermería de manera objetiva y clara de cualquier incidente ocurrido durante la etapa previa al vuelo^(3,4)^. Ante los riesgos inherentes al traslado aéreo, los cuidados relacionados con la sujeción del paciente pediátrico son esenciales para evitar el agravamiento del cuadro clínico. La sujeción del niño al asiento de la aeronave favorece la seguridad del paciente y permite al equipo la identificación temprana de alteraciones clínicas, como crisis convulsivas, vómitos o cambios en la coloración de la piel. La combinación de las correas del ked, las correas de araña de la camilla rígida y los cinturones de seguridad de la aeronave proporciona mayor estabilidad, minimizando los movimientos bruscos durante el vuelo y contribuyendo a la integridad física y psíquica del paciente pediátrico^(4)^.

Durante el vuelo, el médico y el enfermero se colocan de rodillas en el piso de la aeronave debido al espacio físico reducido y a la limitación de movilidad. De esta manera, los procedimientos de soporte vital avanzado, como la intubación orotraqueal, las compresiones torácicas, la desfibrilación cardíaca y el drenaje torácico, se planifican previamente al embarque.

El protocolo establece que el traslado aéreo del paciente pediátrico sin ventilación mecánica debe realizarse por la puerta izquierda de la aeronave, con entrada en posición cefálica hasta la acomodación caudal completa. La elección de la puerta izquierda en esta situación se justifica por la configuración interna de la aeronave, que favorece el posicionamiento de la cabecera del paciente orientada hacia el equipo de asistencia, lo que permite un mejor acceso a las vías respiratorias y a la monitorización durante el vuelo.

Tras la entrada, se procede a la fijación de los cinturones de seguridad y al anclaje de la tabla rígida, junto con los equipos necesarios, según la edad y el peso del niño. En niños con un peso aproximado de 7 kg y menores de 1 año, el protocolo recomienda el uso del KED pediátrico. Para niños mayores de un año y con un peso superior a 7 kg, el protocolo indica el uso del dispositivo para adultos, junto con la camilla rígida para el traslado hasta la aeronave^(4)^.

Para los pacientes pediátricos en ventilación mecánica, el protocolo orienta que la entrada al vuelo se realice por la puerta derecha de la aeronave, desde la posición caudal hasta la entrada cefálica completa. Esta conducta se justifica por la necesidad de posicionar al paciente con la extremidad cefálica cerca de los equipos de ventilación, facilitando el acceso y el control de los dispositivos durante el vuelo. Cabe destacar que la elección de la puerta de acceso puede requerir adaptaciones según el lugar de aterrizaje y las condiciones del terreno, siendo necesario evaluar, en cada situación, si el paciente debe ser introducido en la aeronave con la cabeza o los pies en dirección a la entrada. Tras la acomodación, se procede a la fijación de los cinturones de seguridad y al anclaje de la tabla rígida en el asiento de la tripulación, así como a la instalación de los equipos de monitoreo. En estos casos, el uso del KED, ya sea para adultos o pediátrico, junto con la tabla rígida, favorece la seguridad durante el traslado y sirve de soporte para la colocación de los equipos de ventilación y monitoreo, reforzando la estabilidad del paciente durante el vuelo^(4)^.

### Cuidados Post-Vuelo

Tras el vuelo, tanto el médico como el enfermero registran y transmiten toda la información y las complicaciones ocurridas antes, durante y después de la llegada del niño al destino de tratamiento. Se procede a la reposición de los insumos y equipos utilizados durante el traslado del paciente pediátrico, así como a la limpieza y desinfección del interior de la aeronave y de los dispositivos de sujeción empleados en el vuelo, asegurando la disponibilidad del servicio para nuevas intervenciones^(3,4)^.

El protocolo elaborado incluye, además, un diagrama de flujo asistencial para la atención del paciente pediátrico, con la descripción de las etapas correspondientes a cada fase de la atención durante el transporte aeromédico (Figura 3).

**Figura 3:** Diagrama de flujo de activación para la atención de pacientes pediátricos. Florianópolis, SC, Brasil, 2025.

## DISCUSIÓN

El transporte aeromédico de pacientes pediátricos en aeronaves de ala giratoria se configura como una práctica de alta complejidad asistencial, en la que la seguridad del paciente depende directamente de la adecuación de los dispositivos de inmovilización y de la organización de la asistencia prestada durante todas las etapas del vuelo. Estudios internacionales evidencian que la variabilidad en las prácticas de inmovilización y sujeción durante el transporte aéreo puede aumentar el riesgo de eventos adversos, especialmente en pacientes pediátricos con condiciones clínicas inestables o en ventilación mecánica^(16,18)^.

En este contexto, la experiencia descrita demuestra que la elaboración de un protocolo de atención específico para la sujeción del paciente pediátrico contribuyó a la estandarización de los procedimientos y a la reducción de la variabilidad de la práctica clínica en el servicio aeromédico. La organización de la atención se llevó a cabo en tres momentos: pre-vuelo, durante el vuelo y post-vuelo, lo que favoreció la planificación anticipada, la mitigación de riesgos y la continuidad de la atención, aspectos considerados esenciales en escenarios caracterizados por restricciones físicas, vibración, ruido y acceso limitado al paciente durante el vuelo^(2,7)^.

En la etapa previa al vuelo, la definición de criterios para la verificación de equipos y la selección de dispositivos de sujeción, de acuerdo con la edad, el peso, la estatura y el estado clínico del niño, resultó fundamental para la seguridad del transporte. La literatura señala que la inadecuación del dispositivo de sujeción al perfil del paciente pediátrico se asocia con una mayor inestabilidad durante el traslado y dificultad para realizar intervenciones de emergencia^(19)^. Así, la sistematización de esta etapa contribuye a la anticipación de las necesidades asistenciales y a una toma de decisiones clínicas más acertada.

Durante el vuelo, la estandarización de la aplicación de los dispositivos de sujeción, priorizando la estabilidad, el mantenimiento de las vías respiratorias y la posibilidad de monitoreo continuo, encuentra respaldo en las recomendaciones internacionales para el transporte aeromédico pediátrico. Las particularidades anatómicas y fisiológicas de esta población, con una mayor proporción cefálica, flexibilidad del esqueleto y mayor vulnerabilidad a la hipotermia, exigen estrategias de inmovilización que garanticen la seguridad sin comprometer la ventilación ni el acceso a las vías respiratorias^(10)^.

El uso del KED asociado a una tabla rígida, tal como se describe en el protocolo, se mostró compatible con estas recomendaciones, al permitir una estabilidad adecuada y el soporte para las intervenciones necesarias durante el vuelo. Otro aspecto relevante se refiere a la posibilidad de utilizar dispositivos alternativos de sujeción, como el asiento de seguridad para vehículos, en situaciones específicas. La evidencia indica que, cuando se fijan adecuadamente, estos dispositivos pueden representar una alternativa segura en el transporte aeromédico, especialmente en niños que presentan una reacción intensa a la inmovilización rígida^(16)^.

Después del vuelo, la estandarización del registro de la información asistencial y de la reorganización operativa del servicio contribuye a la continuidad de la atención y a la seguridad del paciente, además de favorecer la trazabilidad de las conductas adoptadas. La literatura destaca que los registros sistematizados son esenciales para la comunicación entre equipos y para la evaluación de la calidad de la atención en servicios de urgencia y emergencia^(3)^.

Cabe destacar, además, el papel del enfermero de vuelo como profesional central en la organización de la atención y en la gestión de los recursos durante el transporte aeromédico. La actuación del enfermero en la verificación de equipos, en la aplicación de dispositivos de inmovilización y en la toma de decisiones compartida con el equipo médico está en consonancia con las normativas del Consejo Federal de Enfermería, que reconocen su responsabilidad tanto asistencial como gerencial en la atención prehospitalaria e interhospitalaria en vehículo aéreo^(4)^. Los estudios señalan la importancia de la preparación de los profesionales de la salud, ya que realizar procedimientos que salvan la vida de los niños sin las herramientas o el entrenamiento adecuado representa un desafío significativo para el equipo^(20,21)^.

Ante lo expuesto, el protocolo contribuye a la estandarización de la inmovilización del paciente pediátrico durante el traslado aéreo, fortaleciendo la autonomía profesional y respaldando la toma de decisiones clínicas en un escenario de alta complejidad, en el que el tiempo y el espacio imponen limitaciones importantes a la atención. Como limitación del estudio, cabe destacar la escasez de publicaciones científicas dirigidas específicamente a la práctica de la enfermería en la inmovilización del paciente pediátrico durante el traslado aeromédico.

Como contribución al área de la salud, el protocolo elaborado describe los principales cuidados de inmovilización durante el vuelo de pacientes pediátricos, reuniendo información esencial para la seguridad del paciente y presentando potencial de aplicabilidad en otros contextos aeromédicos.

## CONCLUSIÓN

El transporte aeromédico de pacientes pediátricos en aeronaves de ala giratoria expone a los pacientes a riesgos inherentes que pueden interferir significativamente en su estado de salud, lo que exige medidas preventivas y terapéuticas sistematizadas para minimizar las complicaciones durante el traslado. La experiencia descrita evidencia que la elaboración de un protocolo de cuidados enfocado en la inmovilización del paciente pediátrico contribuye a mejorar la calidad de la atención, al sistematizar las conductas y reducir la variabilidad de la atención en un contexto de alta complejidad. El protocolo desarrollado estandarizó las prácticas de inmovilización del paciente pediátrico en el servicio aeromédico Arcanjo, promoviendo la estandarización, la seguridad y el apoyo a la toma de decisiones clínicas durante el vuelo, reduciendo el riesgo de complicaciones secundarias durante el transporte y fortaleciendo la práctica profesional en el ámbito de la atención aeromédica.

## Data Availability

De acuerdo con lo recomendado por la Ciencia Abierta, el enlace al repositorio en el que se depositaron los datos de la investigación: https://repositorio.ufsc.br/xmlui/bitstream/handle/123456789/247267/PGCF0174-D.pdf?sequence=1.
